# Development of a Model to Predict 10-Year Risk of Ischemic and Hemorrhagic Stroke and Ischemic Heart Disease Using the China Kadoorie Biobank

**DOI:** 10.1212/WNL.0000000000200139

**Published:** 2022-04-11

**Authors:** Songchun Yang, Yuting Han, Canqing Yu, Yu Guo, Yuanjie Pang, Dianjianyi Sun, Pei Pei, Ling Yang, Yiping Chen, Huaidong Du, Hao Wang, M. Sofia Massa, Derrick Bennett, Robert Clarke, Junshi Chen, Zhengming Chen, Jun Lv, Liming Li

**Affiliations:** Department of Epidemiology & Biostatistics, School of Public Health, Peking University, Beijing, China; Department of Epidemiology & Biostatistics, School of Public Health, Peking University, Beijing, China; Department of Epidemiology & Biostatistics, School of Public Health, Peking University, Beijing, China; Fuwai Hospital Chinese Academy of Medical Sciences, Beijing, China; Department of Epidemiology & Biostatistics, School of Public Health, Peking University, Beijing, China; Department of Epidemiology & Biostatistics, School of Public Health, Peking University, Beijing, China; Chinese Academy of Medical Sciences, Beijing, China; Medical Research Council Population Health Research Unit at the University of Oxford; Clinical Trial Service Unit & Epidemiological Studies Unit, Nuffield Department of Population Health, University of Oxford, UK; Peking University Center for Public Health and Epidemic Preparedness & Response, Beijing, China; Medical Research Council Population Health Research Unit at the University of Oxford; Clinical Trial Service Unit & Epidemiological Studies Unit, Nuffield Department of Population Health, University of Oxford, UK; Medical Research Council Population Health Research Unit at the University of Oxford; Clinical Trial Service Unit & Epidemiological Studies Unit, Nuffield Department of Population Health, University of Oxford, UK; NCDs Prevention and Control Department, Zhejiang CDC, Hangzhou, China; Clinical Trial Service Unit & Epidemiological Studies Unit, Nuffield Department of Population Health, University of Oxford, UK; Clinical Trial Service Unit & Epidemiological Studies Unit, Nuffield Department of Population Health, University of Oxford, UK; Clinical Trial Service Unit & Epidemiological Studies Unit, Nuffield Department of Population Health, University of Oxford, UK; China National Center for Food Safety Risk Assessment, Beijing, China; Clinical Trial Service Unit & Epidemiological Studies Unit, Nuffield Department of Population Health, University of Oxford, UK; Department of Epidemiology & Biostatistics, School of Public Health, Peking University, Beijing, China; Peking University Center for Public Health and Epidemic Preparedness & Response; Key Laboratory of Molecular Cardiovascular Sciences (Peking University), Ministry of Education, Beijing, China; Department of Epidemiology & Biostatistics, School of Public Health, Peking University, Beijing, China; Peking University Center for Public Health and Epidemic Preparedness & Response, Beijing, China

## Abstract

**Background and Objectives:**

Contemporary cardiovascular disease (CVD) risk prediction models are rarely applied in routine clinical practice in China due to substantial regional differences in absolute risks of major CVD types within China. Moreover, the inclusion of blood lipids in most risk prediction models also limits their use in the Chinese population. We developed 10-year CVD risk prediction models excluding blood lipids that may be applicable to diverse regions of China.

**Methods:**

We derived sex-specific models separately for ischemic heart disease (IHD), ischemic stroke (IS), and hemorrhagic stroke (HS) in addition to total CVD in the China Kadoorie Biobank. Participants were age 30–79 years without CVD at baseline. Predictors included age, systolic and diastolic blood pressure, use of blood pressure–lowering treatment, current daily smoking, diabetes, and waist circumference. Total CVD risks were combined in terms of conditional probability using the predicted risks of 3 submodels. Risk models were recalibrated in each region by 2 methods (practical and ideal) and risk prediction was estimated before and after recalibration.

**Results:**

Model derivation involved 489,596 individuals, including 45,947 IHD, 43,647 IS, and 11,168 HS cases during 11 years of follow-up. In women, the Harrell C was 0.732 (95% CI 0.706–0.758), 0.759 (0.738–0.779), and 0.803 (0.778–0.827) for IHD, IS, and HS, respectively. The Harrell C for total CVD was 0.734 (0.732–0.736), 0.754 (0.752–0.756), and 0.774 (0.772–0.776) for models before recalibration, after practical recalibration, and after ideal recalibration. The calibration performances improved after recalibration, with models after ideal recalibration showing the best model performances. The results for men were comparable to those for women.

**Discussion:**

Our CVD risk prediction models yielded good discrimination of IHD and stroke subtypes in addition to total CVD without including blood lipids. Flexible recalibration of our models for different regions could enable more widespread use using resident health records covering the overall Chinese population.

**Classification of Evidence:**

This study provides Class I evidence that a prediction model incorporating accessible clinical variables predicts 10-year risk of IHD, IS, and HS in the Chinese population age 30–79 years.

Cardiovascular diseases (CVD), including ischemic heart disease (IHD), ischemic stroke (IS), and hemorrhagic stroke (HS), are the leading causes of the global burden of diseases.^1^ Risk prediction models are important tools for identifying high-risk individuals for early interventions in the primary prevention of CVD. Although the availability of many models has prompted researchers to focus on tailoring and improving existing models in local populations,^2^ there is currently no model that simultaneously has the following characteristics and can be widely used in the Chinese population.

The first is to distinguish the risks between IS and HS. The Chinese population has a much higher incidence of HS than Western populations.^1^ However, currently recommended models worldwide, including China, either did not include HS as the outcome^3,4^ or did not distinguish IS from HS.^5–9^

The second is to achieve efficient and widespread use of the risk prediction model by linking to dynamic electronic health records.^3,6,10–12^ In China, establishing resident health records (RHRs) has been one of the basic services provided by the National Basic Public Health Service Program (NBPHSP) since 2009, covering the whole population in the Chinese mainland. The free RHRs for the whole population include sociodemographic information, personal and family medical history, lifestyle, and noninvasive physical examinations (e.g., height, weight, waist circumference [WC], and blood pressure). Free blood cholesterol measurements are only provided to people ≥65 years of age. However, previous models mostly rely on blood lipids.^3–7,9^

The third is to be widely applicable to different regions. Risk factors for different CVD subcategories may vary or have different effect estimates.^13–15^ For a model that predicts the risk of composite outcome (single-model estimated, such as total CVD), the magnitude of association (i.e., regression coefficient in the model) might be affected by the composition proportion of each CVD subcategory in the derivation dataset, leading to potentially biased estimations. Therefore, considering that the absolute risks of 3 CVD subcategories vary substantially among different regions in China,^16^ previous risk prediction models based on single-model approach are less applicable to broad areas of China.^3–7,9^ To address this issue, the WHO CVD Risk Chart Working Group has proposed a new modeling and recalibration approach to help adapt the original models to different regions.^8^

Based on the China Kadoorie Biobank (CKB), one of the largest population-based cohorts globally, the present report primarily aimed to develop pragmatic disease-specific and overall CVD risk prediction models based on widely available variables in RHRs of China, covering IHD, IS, and HS. Next, we evaluated 2 model recalibration approaches based on the method proposed by the WHO CVD Risk Chart Working Group to adapt our models to different regions. We further evaluated the potential incremental value of more predictors to the derived models.

## Methods

### Study Population

CKB is an ongoing prospective study of 512,725 participants age 30 to 79 years who were enrolled from 5 urban and 5 rural regions situated in the northeast, northwest, east, south, and southwest of China during 2004–2008. The study regions were selected according to local disease patterns, exposure to risk factors, population stability, quality of death and disease registries, and local commitment and capacity. Within each study region, all potentially eligible participants in each of 100–150 rural villages or urban residential committees were invited to take part in the survey. The estimated population response rate was about 30% (26%–38% in the 5 rural regions and 16%–50% in the 5 urban regions). Details of the study have been described elsewhere.^17^ Briefly, all participants had valid baseline data, including a complete interviewer-administered laptop-based questionnaire and physical measurements conducted by trained health workers using calibrated instruments and standard protocols. A 10-mL random blood sample was collected for each participant with the time of the last meal recorded.

### Study Design

**Figure 1:**
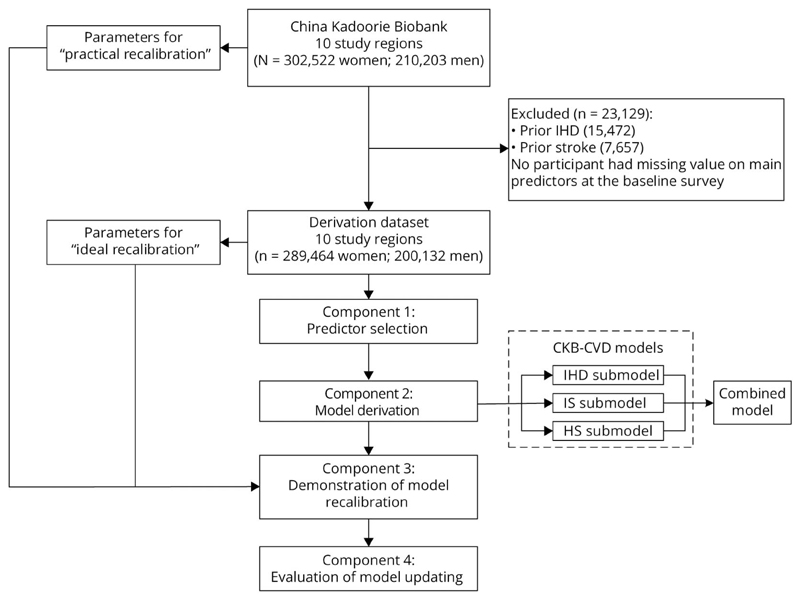
Study Overview HS = hemorrhagic stroke; IHD = ischemic heart disease; IS = ischemic stroke.

In this study, 4 interrelated components were involved (Figure 1): (1) predictor selection—we selected several predictors from a predefined list of candidate variables for model derivation; (2) model derivation—we then derived sex-specific and outcome-specific 10-year CVD risk prediction models among participants without CVD at baseline; namely, the CKB-CVD models; (3) demonstration of model recalibration—to support the flexible updating of the derived models, we compared 2 methods of recalibration; (4) evaluation of model updating—based on the derived models, we further evaluated the predictive utility of CVD risk factors that were not included in the model derivation process.

### Assessment of Candidate Predictors

We predefined 16 candidate predictors (eTable 1, links.lww.com/WNL/B915) for model derivation according to the following criteria: (1) established or probable risk factors of CVD; (2) widely available in RHRs; and (3) available in the CKB baseline survey. Other CVD risk factors that are not widely available in RHRs (eTable 1) were used to explore the ability to improve the derived models.

The baseline questionnaire collected information on socio-demographic characteristics, lifestyle behaviors, dietary habits, and personal and family medical history. All participants were asked "How often do you smoke tobacco now?"; the 4 response options were (1) do not smoke now, (2) only occasionally, (3) on most days, and (4) every day. Current daily smokers were defined as those who answered (4). Systolic blood pressure (SBP) and diastolic blood pressure (DBP) at baseline were measured using a UA-779 digital sphygmomanometer. Measurements were made after a minimum of 5 minutes of sitting and repeated twice. If the 2 measurements for SBP were more than 10 mm Hg apart, a third measurement was taken. Only the last 2 readings were recorded and averaged for further analyses. Use of blood pressure-lowering treatment was self-reported by participants and was defined as answering "yes" to "Is your diagnosed hypertension still on treatment?" or "Did you take any drugs to lower blood pressure in the last 2 days?" History of diabetes was defined as self-reported diabetes diagnosed by a physician before the baseline survey. We did not use random blood glucose measurements as our previous study did because of its limited availability in China.^18^ WC was measured midway between the iliac crest and the lower rib margin at the end of normal expiration using a flexible plastic tape to the nearest 0.1 cm. The assessment and definition of other candidate predictors are shown in eTable 1 (links.lww.com/WNL/B915).

### Ascertainment of Outcomes

All participants were followed up for incident disease outcomes since their enrollment at baseline. Incident events were identified by using linkages with local disease and death registries and the national health insurance system and supplemented by active follow-up.^17^ The loss to follow-up was <1% prior to censoring on December 31, 2017. Trained staff blinded to baseline information coded all events using ICD-10. In this study, IHD events included fatal or nonfatal angina (I20), myocardial infarction (I21–I23), and other IHD (I24–I25); IS events included fatal or nonfatal cerebral infarction (I63); HS events included fatal or nonfatal subarachnoid hemorrhage (SAH) or intracerebral hemorrhage (ICH) (I60–I62). Unspecified stroke events were coded as I64. The total CVD was defined as fatal or nonfatal IHD and stroke (I20–I25, I60–I64). Since 2014, medical records of incident IHD and stroke cases have been retrieved and reviewed by qualified cardiovascular specialists blinded to baseline exposures of patients. By October 2018, of the retrieved medical records of 33,515 IHD cases, 34,758 IS cases, and 5023 HS cases, the confirmed rates of the diagnosis were 87.9%, 91.5%, and 80.4% for IHD, IS, and HS, separately.

### Statistical Analysis

Cox proportional hazard models were used to develop prediction models, stratified by 10 study regions and with time on study as the time scale. Time on study was the time from the baseline to the first of the following: first diagnosis of CVD, death, loss to follow-up, or December 31, 2017. All analyses were performed separately for women and men. Discrimination performance was accessed by the Harrell C statistic.^19^ Calibration performance was assessed graphically by comparing the mean predicted risks at 10 years with the observed risks across deciles of predicted risks. The Nam-D’Agostino test was used to quantify the agreement of fit.^20^

In the predictor selection component, the basic model included 4 well-established CVD risk factors as predictors: baseline age (years), SBP (mm Hg), current daily smoking (yes or no), and history of diabetes (yes or no). Interactions between age and the other 3 predictors were also included.^8^ Based on the relative integrated discrimination improvement (IDI) in the total CVD risk prediction model,^21^, 3 additional predictors—use of blood pressure-lowering treatment (yes or no), DBP (mm Hg), and WC (cm)—were also selected for model derivation (eMethods and eTables 2 and 3, links.lww.com/WNL/B915).

In the model derivation component, we developed models separately for IHD, IS, and HS (submodel of the CKB-CVD models; Figure 1) to allow separate recalibration to the disease-specific incidences in different regions. Predictors included the afore-mentioned 7 variables and interactions between age and the other 6 variables. Before deriving the models, the necessity of logarithmic transformation of all continuous variables was examined, because the natural logarithm is the most common way to handle continuous variables in previous risk prediction models.^5,7^ Finally, all continuous variables were modeled as simple linear form and were centered (age at 55 years, SBP at 120 mm Hg, DBP at 80 mm Hg, and WC at 80 cm) to provide a more straightforward interpretation of the regression estimates and facilitate recalibration ofthe models.^3,6,8^ The proportional hazards assumption was assessed by plotting the scaled Schoenfeld residuals vs time. Either nonexistent or minimal deviations were observed. Baseline survival estimate at 10 years (S_0_[10]) was estimated by pooling the S_0_(10) across regions weighted by the number of cases that had occurred by 10 years.^8^ To avoid overfitting, we used an internal-external cross-validation approach in which each region was left out of the model derivation and used to calculate a validation Harrell C in turn.^22^ The total CVD risk was combined in terms of conditional probability using the predicted risks calculated by the 3 submodels (combined model): that is, *Risk_CVD_* = 1 – (1 – *Risk_IHD_)* x (1 – *Risk_IS_*) ^x^ (1 – *Risk_HS_*).^8^ We checked the assumption of independence among risks of the 3 CVD subcategories by considering competing risks.^8^

To simulate the process of applying the CKB-CVD models in different regions, model recalibration was performed separately for 10 study regions. We calculated the ideal performance metrics assuming the availability of observed 10-year risks in each study region (ideal recalibration). However, in practice, the observed 10-year risks of the target population are difficult to obtain. The recalibration process could also use age-specific (5-year age groups) and sex-specific mean risk factor levels and annual incidence estimates of CVD in each study region (practical recalibration) (eMethods, links.lww.com/WNL/B915).^8^ Model performance before and after recalibration was evaluated in the whole derivation dataset and each study region. We also evaluated model performance before and after recalibration in participants <65 and ≥65 years of age, with and without hypertension, and with and without diabetes.

We further explored whether the remaining predictors (eTable 1, links.lww.com/WNL/B915) could be used for updating CKB-CVD models in the future. We added each predictor individually and evaluated its relative IDI for each major CVD subtype, as we did in the predictor selection component outlined above.

This study adhered to the TRIPOD (Transparent Reporting of a Multivariable Prediction Model for Individual Prognosis or Diagnosis) statement for reporting.^23^ Analyses were done with Stata 15.0. Figures were produced using R 3.6.0.

### Standard Protocol Approvals, Registrations, and Patient Consents

CKB had ethical approvals from the Ethical Review Committee of the Chinese Center for Disease Control and Prevention (Beijing, China) and the Oxford Tropical Research Ethics Committee, University of Oxford (UK). All participants provided a written informed consent form.

## Results

### Overview of the Study Population

The study population included 302,522 women and 210,203 men age 30–79 years at the baseline survey. Participants who had been diagnosed with IHD (n = 15,472) or stroke (n = 7,657) before the baseline survey were excluded from the derivation dataset (Figure 1). No participant had missing data on the main predictors used in the model derivation. The remaining 489,596 participants had a median age of 52 years (25th–75th percentile 43–60) for women and 50 years (42–58) for men (Table 1). During a median of 11 years of follow-up, 86,464 (17.7%) participants had CVD events, including 45,947 IHD, 43,647 IS, and 11,168 HS events. The standardized 10-year risks of 3 CVD subcategories varied greatly across regions (eFigure 1, links.lww.com/WNL/B915).

### The Newly Derived CKB-CVD Models

**Figure 2:**
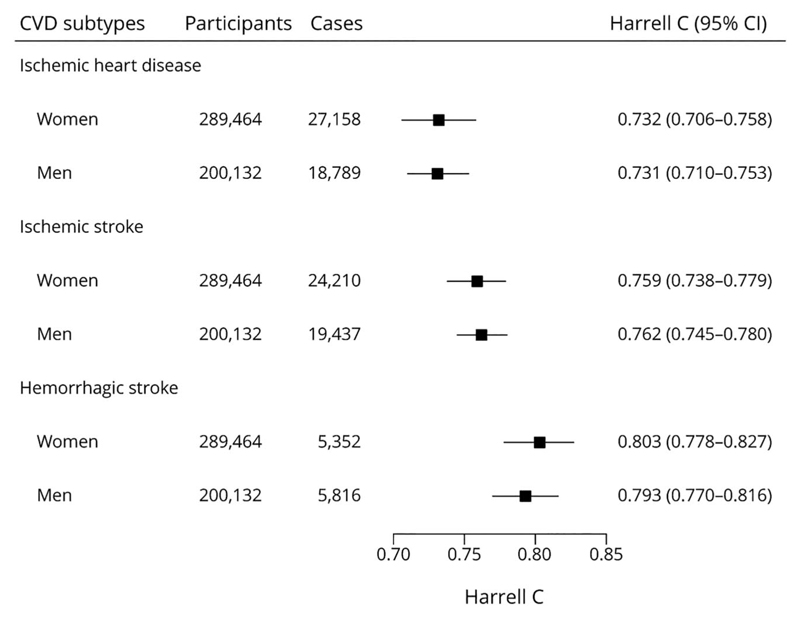
Harrell C Statistics of the CKB-CVD Models Harrell C was calculated using an internal–external cross-validation approach in which each study region was left out of the model fit and used in validation in turn. Harrell C shown is the result of pooling Harrell C from each external study region. CKB = China Kadoorie Biobank; CVD = cardiovascular diseases.

Seven predictors were used to derive the sex-specific CKB-CVD models separately for 3 CVD subcategories (Table 2). The adjusted hazard ratios (HRs) of predictors varied across different sexes and different CVD subcategories. For example, HRs of DBP (per 10 mm Hg) ranged from 1.05 (95% CI 1.04–1.07) for IHD in women to 1.33 (1.29–1.38) for HS in men; HRs ofWC (per 10 cm) ranged from 0.92 (0.89–0.95) for HS in women to 1.16 (1.14–1.17) for IHD in women. Regression coefficients for each predictor are provided in eAppendix 1 (links.lww.com/ WNL/B916). An example risk calculation is shown in eMethods. The assumption of independence among risks of 3 CVD subcategories was not violated (eFigure 2). The HS submodel had the best discrimination performance in internal validation (Figure 2). Calibration performances of the 3 submodels were poor in most study regions (eFigure 3). In sensitivity analysis, we derived models separately for ICH, whose beta coefficients were similar to those of the HS models (eTable 4).

**Figure 3:**
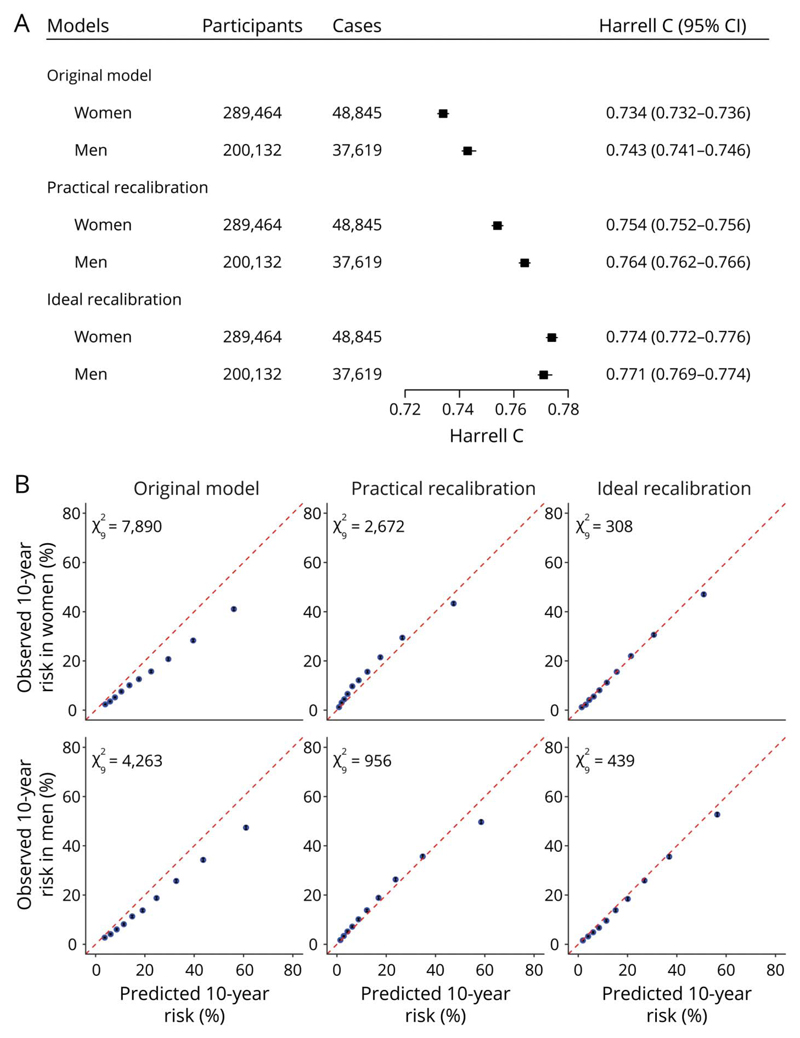
Model Performance of the Combined Model Before and After Recalibration The original model refers to the combined model before recalibration. (A) Discrimination performance. (B) Calibration performance. $\chi_{9}^{2}$ is the Nam-D’Agostino test χ^2^ with 9 degrees of freedom. The 95% CIs of the observed 10-year risk (black error bar) were too narrow to display in the calibration plots clearly.

As for the combined model before recalibration (original model in the following), Harrell C was 0.734 (95% CI 0.732–0.736) for women and 0.743 (0.741–0.746) for men in the derivation dataset (Figure 3), ranging from 0.705 (0.701–0.710) for women in Harbin to 0.804 (0.795–0.813) for women in Suzhou (eFigure 4, links.lww.com/WNL/B915). Calibration was poor in the derivation dataset (Figure 3) and most study regions (eFigure 5).

### Recalibration of the CKB-CVD Models

After practical recalibration, Harrell C increased to 0.754 (95% CI 0.752–0.756) for women and 0.764 (0.762–0.766) for men, and calibration improved for both sexes in the derivation dataset. Models after ideal recalibration showed the best model performances (Figure 3). Harrell C in each study region almost remained unchanged after practical recalibration and ideal recalibration (data not shown). Calibration performances improved in almost all study regions after ideal recalibration and in half of the study regions after practical recalibration. The number of urban regions with improved calibration performance after practical recalibration was greater than those in rural regions (eFigure 5, links.lww.com/WNL/B915). An example of practical recalibration is shown in eMethods. We also provide an interactive 10-year CVD risk calculator and a calculator of practical recalibration measures in eAppendix 1 (links.lww.com/WNL/B916).

Discrimination of the original model was lower in the patients who were older (≥65 years), had hypertension, or had diabetes, especially in older women (Harrell C 0.584, 95% CI 0.579–0.589) (eFigure 6, links.lww.com/WNL/B915). Separate recalibration in each study region improved the discrimination and the calibration among these participants (eFigures 7–9).

### Evaluation of Model Updating

**Figure 4:**
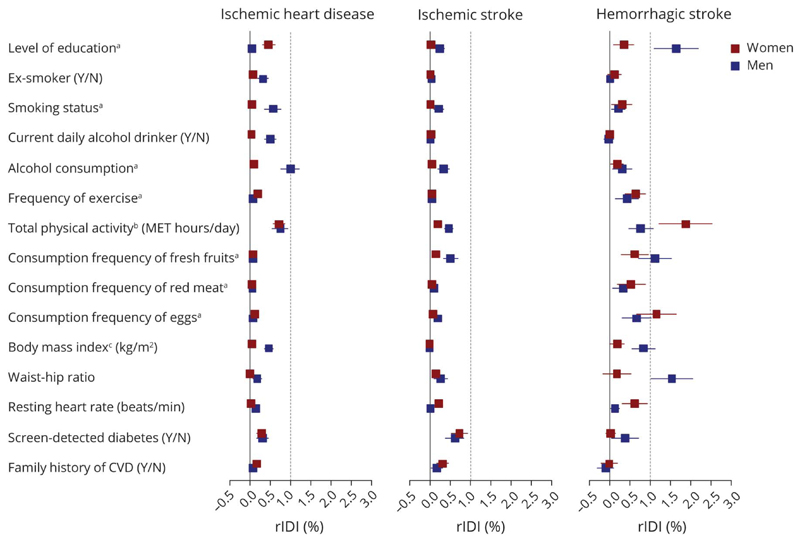
Relative Integrated Discrimination Improvement of Other Predictors Based on the CKB-CVD Models The relative integrated discrimination improvement (rIDI) (%) was calculated with the same method used in the predictor selection component (eMethods). ^a^Detailed categories of multicategorical variables are as follows: level of education (6 groups: no formal school, primary school, middle school, high school, technical school or college, and university); smoking status (5 groups: nonsmoker, former smoker, current smoker who smoked <10,10–19, or ≥20 cigarettes or equivalents per day); alcohol consumption (7 groups: nondrinker, former drinker, weekly drinker, daily drinker with an intake of <15,15–29,30–59, or ≥60 g/d of pure alcohol); frequency of exercise (4 groups: never or rarely, 1–3 times/month, 1–5 times/week, and daily or almost daily); consumption frequency of fresh fruits, red meat, and eggs (5 groups: daily, 4–6 d/wk, 1–3 d/wk, monthly, and never or rarely). ^b^The total physical activity was natural log-transformed before analysis. ^c^Two participants had missing value of body mass index and were excluded from the current analysis. CKB = China Kadoorie Biobank; CVD = cardiovascular diseases; MET = metabolic equivalent; rIDI = relative integrated discrimination improvement.

Based on the CKB-CVD models, only level of education and waist-hip ratio in men and total physical activity in women had relative IDI greater than 1% for the HS submodel. Other predictors had a small or no effect on improving the 3 submodels (Figure 4).

### Classification of Evidence

This study provides Class I evidence that a prediction model incorporating accessible clinical variables predicts 10-year risk of IHD, IS, and HS in the Chinese population 30–79 years of age.

## Discussion

In this large population-based Chinese cohort, we developed pragmatic sex-specific risk models that predict 10-year risks of CVD subcategories and overall CVD. Given the high burden of HS in the Chinese population, our models distinguish stroke subtypes and should be more applicable to the Chinese than previous models. Our models achieved good discrimination of risk groups, even without using blood lipids information, indicating a potentially wider use based on RHRs. Flexible model updating methods improved the model performance when used in different regions.

Two CVD primary prevention guidelines for Chinese individuals have recommended two 10-year CVD risk prediction models as risk assessment tools: one derived from the Chinese Multi-provincial Cohort Study (CMCS) and the other derived from the China-PAR Project.^25,26^ The loss to follow-up in both cohorts, 21.3%^27^ and 9.9%,^7^ respectively, may increase the risk of bias according to PROBAST.^28^ Also, both models relied on blood lipids, used hard IHD as the outcome, and did not consider the risk of HS separately.^4,7^ Few studies have derived risk prediction models for HS. One study was performed in a small cohort of 4,400 Chinese steelworkers recruited before 1980, with only 33 HS events in the derivation dataset.^29^ The other study was conducted using 3 population-based cohorts from the United States and the Netherlands and included 325 intracerebral hemorrhage (ICH) events.^30^ As for IHD, previous studies have reported that patients with angina also have a poor prognosis.^31^ The simultaneous risk prediction of hard IHD and soft stroke might lead to misunderstandings among model users. Therefore, consistent with QRISK3 and PREDICT,^3,6^ we included such outcomes in high-risk population screening.

Most previous models only included SBP as the predictor. In the current study, the addition of DBP to the basic model for HS substantially improved the predictive ability. Although the relative IDI for the IHD and IS submodels was small, the category-free net reclassification improvement was >10% for IS and >5% for IHD in both sexes (data not shown). Body mass index was included as a predictor in the WHO non–laboratory-based model.^8^ However, it did not improve the predictive ability as much as WC in our analysis (the predictor selection component), consistent with the finding of the China-PAR Project.^7^ We therefore included DBP and WC as predictors, given their incremental values in outcome prediction and wide availability in RHRs of China. Our models used self-reported history of diabetes as a predictor, consistent with the practice of RHRs. Despite nearly half of the undiagnosed diabetes cases being excluded in our cohort, the addition of screen-detected diabetes only led to a minimal improvement of the 3 submodels.

Older individuals and individuals with hypertension or diabetes are major users of health services in China. We examined model performances stratified by these characteristics and found models had the lowest discrimination among individuals age ≥65 years. This is mostly due to the lower contribution of age to the models when the analysis was limited to older people. Previous models for older people, using traditional cardiovascular risk factors as predictors, also usually had Harrell C below 0.65.^32^ Because additional free blood tests are available for the elderly in the NBPHSP, there is a promise of improving risk prediction for the elderly by adding more predictors.

There were significant differences in the calibration performances of the original models across different study regions, including both the 3 submodels and the combined CVD risk model. This finding suggests that previous universal CVD risk prediction models may encounter the same problems when applied to different regions of China, highlighting the importance of model recalibration. The ideal recalibration is not easy to achieve because long-term follow-up is required to obtain the observed 10-year risks. The WHO CVD Risk Chart Working Group provided a flexible recalibration method based on cross-sectional population data (i.e., practical recalibration in our study).^8^ The current study suggests that the recalibration method proposed by WHO is effective. However, there was still a gap between practical recalibration and ideal recalibration. One possible explanation is that the number of participants in each 5-year age group was relatively small for each study region, leading to errors in estimating the mean risk factor levels and annual incidence of diseases. We assume that if a large and representative population was used to estimate the recalibration measures, the effect of practical recalibration would be closer to that of ideal recalibration. However, further studies are warranted.

This study population was uniquely large. The loss to follow-up was <1% prior to the global censoring date. More than 100,000 CVD events were documented during a follow-up of 11 years. Unspecified stroke accounted for only 1.4% and 1.2% of first stroke events in women and men. These advantages allowed us to derive risk prediction models separately for 3 CVD subcategories and obtain robust coefficient estimates. Our models have the potential to be widely used because of readily available predictors from the RHRs of China. The recalibration approach employed in this study enables the flexible updating of our models in different regions in China. We have provided a calculator of practical recalibration measures (eAppendix 1, links. lww.com/WNL/B916) and statistical codes required to calculate our models^33^ to support model updating.

Several limitations merit consideration. First, independent samples for external validation of our models are best from local populations in which our models are recalibrated to their CVD incidences and risk factor levels and evaluated the predictive performance. However, the lack of other regional cohorts of middle-aged and older adults with sufficiently large sample size and including >10 years of follow-up in China limits the options for an effective external validation. With the development of more regional cohorts launched by the China Precision Medicine Initiative and regional electronic RHRs system since the mid-2010s, further validation and updating of our models are warranted. Second, as a population-based cohort that aimed to follow up for a long time, CKB was not designed to be representative of the general population in China. Despite this, the inclusion of a considerable number of participants from different regions across China and with diverse sociodemographic characteristics enables the study to provide robust regression coefficients and be generalizable to broad populations. When the models are applied to other regions, a recalibration process based on local disease rates and risk factor levels helps improve the applicability of the models. Third, SAH accounts for only a small proportion of all HS cases in the Chinese population,^34^ with the corresponding proportion of <10% in CKB, precluding us from deriving robust models separately for SAH.^35^ We chose to present the HS models that did not distinguish between ICH and SAH in our primary analysis because the coefficients for ICH-specific models hardly ever changed.

Based on a large population-based cohort of Chinese adults, we developed 10-year risk prediction models for IHD, IS, HS, and their combined outcome that may be more widely applicable to RHRs in the NBPHSP of China. This study took account of the high burden of HS and substantial regional differences in absolute risks of major CVD types within China and derived models that can be adapted for different regions of the Chinese population.

## Supplementary Material

Appendix

## Figures and Tables

**Table 1:** Summary of Available CKB Data Used in the Model Derivation

	Women	Men
**Baseline characteristics**		
**Total participants**	289,464	200,132
**Rural area**	163,410 (56.5)	114,776 (57.4)
**Age, y**	50.4 (42.3–58.1)	51.8 (43.1–60.3)
**Current daily smoker**	5,874 (2.0)	114,218 (57.1)
**Use of blood pressure–lowering treatment**	34,062 (11.8)	20,388 (10.2)
**Systolic blood pressure, mm Hg**	126.0 (114.0–141.0)	129.5 (119.0–143.0)
**Diastolic blood pressure,mmHg**	76.0 (69.0–83.0)	78.5 (71.5–86.0)
**Waist circumference, cm**	78.2 (72.0–84.9)	81.3 (74.5–88.5)
**Self-reported diabetes**	8,258 (2.9)	5,055 (2.5)
**Screen-detected diabetes**	7,933 (2.7)	5,169 (2.6)
**Cardiovascular outcomes**		
**Total person-years observed^a^**	3,185,178	2,137,503
**Follow-up time,^a^ y**	11.2 (10.3–12.2)	11.1 (10.1–12.1)
**Participants with follow-up ≥10 years^a^**	241,127 (83.3)	156,488 (78.2)
**Incident events during the first 10 years/total follow-up period^b^**		
**Ischemic heart disease**	22,765/27,158	15,907/18,789
**Hemorrhagic stroke**	4,482/5,352	4,998/5,816
**Ischemic stroke**	19,752/24,210	16,398/19,437
**Unspecified stroke^c^**	1,199/1,522	1,112/1,344
**Total cardiovascular disease^d^**	41,152/48,845	32,342/37,619

Abbreviation: CKB = China Kadoorie Biobank. Data are n (%) or median (25–75th percentile) unless otherwise specified.

a Person-years were calculated as the time from the baseline date to the first of the following: death, loss to follow-up, or the global censoring date (December 31, 2017).

b Only the first event was included.

c The number of unspecified stroke events as the first stroke events: 434 in women and 337 in men.

d Total cardiovascular disease events included ischemic heart disease events (fatal and nonfatal I20–I25) and stroke events (fatal or nonfatal I60–I64).

**Table 2:** Adjusted Hazard Ratios for Predictors in the CKB-CVD Models

	Women		Men	
	Main effect	Age interaction	Main effect	Age interaction
**Ischemic heart disease**					
**Age per 5 years**	1.40 (1.38–1.41)	—	1.43 (1.41–1.45)	—
**SBP per 10 mm Hg**	1.02 (1.01–1.03)	1.00 (0.99–1.00)	1.05 (1.04–1.07)	1.00 (1.00–1.01)
**DBP per 10 mm Hg**	1.05 (1.04–1.07)	0.99 (0.99–1.00)	1.10 (1.07–1.12)	0.97 (0.96–0.97)
**Use of blood pressure–lowering treatment**	1.55 (1.49–1.61)	0.95 (0.93–0.97)	1.59 (1.52–1.67)	0.97 (0.95–0.99)
**Current daily smoker**	1.17 (1.08–1.28)	0.97 (0.93–1.00)	1.22 (1.19–1.26)	0.98 (0.97–1.00)
**Self-reported diabetes**	1.59 (1.49–1.69)	0.94 (0.92–0.97)	1.82 (1.69–1.95)	0.91 (0.88–0.94)
**Waist circumference per 10 cm**	1.16 (1.14–1.17)	0.97 (0.96–0.98)	1.13 (1.11–1.15)	0.97 (0.96–0.98)
**Baseline survival estimate at 10 years^a^**	0.900	—	0.929	—
**Ischemic stroke**					
**Age per 5 years**	1.45 (1.43–1.46)	—	1.52 (1.50–1.54)	—
**SBP per 10 mm Hg**	1.09 (1.08–1.10)	0.98 (0.97–0.98)	1.13 (1.12–1.15)	0.98 (0.97–0.98)
**DBP per 10 mm Hg**	1.11 (1.09–1.13)	0.99 (0.99–1.00)	1.15 (1.13–1.17)	0.98 (0.98–0.99)
**Use of blood pressure–lowering treatment**	1.54 (1.48–1.60)	0.96 (0.94–0.97)	1.59 (1.52–1.67)	0.94 (0.93–0.96)
**Current daily smoker**	1.30 (1.18–1.42)	0.93 (0.89–0.96)	1.29 (1.24–1.33)	0.97 (0.96–0.99)
**Self-reported diabetes**	1.82 (1.71–1.94)	0.90 (0.87–0.93)	1.83 (1.71–1.97)	0.94 (0.91–0.97)
**Waist circumference per 10 cm**	1.11 (1.09–1.12)	0.98 (0.97–0.98)	1.08 (1.06–1.10)	0.99 (0.99–1.00)
**Baseline survival estimate at 10 years^a^**	0.910	—	0.928	—
**Hemorrhagic stroke**				
**Age per 5 years**	1.51 (1.47–1.54)	—	1.51 (1.47–1.55)	—
**SBP per 10 mm Hg**	1.16 (1.14–1.18)	0.98 (0.97–0.99)	1.18 (1.16–1.21)	0.99 (0.98–1.00)
**DBP per 10 mm Hg**	1.32 (1.28–1.37)	0.96 (0.95–0.98)	1.33 (1.29–1.38)	0.95 (0.94–0.97)
**Use of blood pressure–lowering treatment**	1.62 (1.50–1.74)	0.96 (0.92–0.99)	1.48 (1.37–1.61)	0.95 (0.92–0.99)
**Current daily smoker**	1.28 (1.03–1.59)	0.97 (0.89–1.06)	1.10 (1.04–1.17)	1.00 (0.97–1.02)
**Self-reported diabetes**	1.50 (1.28–1.75)	0.95 (0.89–1.03)	1.45 (1.22–1.72)	0.96 (0.89–1.04)
**Waist circumference per 10 cm**	0.92 (0.89–0.95)	0.99 (0.98–1.01)	0.92 (0.89–0.95)	0.98 (0.97–0.99)
**Baseline survival estimate at 10 years^a^**	0.986	—	0.983	—

Abbreviations: CKB = China Kadoorie Biobank; CVD = cardiovascular diseases; DBP = diastolic blood pressure; SBP = systolic blood pressure.

Predictor variables were centered as follows: age at 55 years, SBP at 120 mm Hg, DBP at 80 mm Hg, and waist circumference at 80 cm.

a The Cox models were stratified by 10 regions. Baseline survival estimate at 10 years (S_0_[10]) was estimated by pooling the S_0_(10) across regions weighted by the number of events by 10 years.

## Data Availability

Cohort description and questionnaires are available online.^24^ Statistical code is available from the corresponding author (J.L.). Details on how to access China Kadoorie Biobank data and details of the data release schedule are available online.^24^

## Glossary

### Abbreviations

CKB: China Kadoorie Biobank
CVD: cardiovascular diseases
DBP: diastolic blood pressure
HR: hazard ratio
HS: hemorrhagic stroke
ICD-10: International Classification of Diseases, Tenth Revision
ICH: intracerebral hemorrhage
IDI: integrated discrimination improvement
IHD: ischemic heart disease
IS: ischemic stroke
NBPHSP: National Basic Public Health Service Program
RHR: resident health record
SAH: subarachnoid hemorrhage
SBP: systolic blood pressure
WC: waist circumference
