# Supplementary material for: Development of a Model to Predict 10-Year Risk of Ischemic and Hemorrhagic Stroke and Ischemic Heart Disease Using the China Kadoorie Biobank
Source: Neurology. Author manuscript; Available in PMC 2022 Jun 22. (PMC9202526; doi:10.1212/WNL.0000000000200139)
Supplement: Appendix [file EMS145999-supplement-Appendix.pdf]

Appendix 1 Authors

Name	Location	Contribution
**Songchun Yang, PhD candidate**	Department of Epidemiology & Biostatistics, School of Public Health, Peking University, Beijing, China	Drafting/revision of the manuscript for content, including medical writing for content; Analysis or intepretation of data
**Yuting Han, PhD candidate**	Department of Epidemiology & Biostatistics, School of Public Health, Peking University, Beijing, China	Analysis or intepretation of data
**Canqing Yu, PhD**	Department of Epidemiology & Biostatistics, School of Public Health, Peking University; Peking University Center for Public Health and Epidemic Preparedness & Response, Beijing, China	Major role in the acquisition of data; Analysis or intepretation of data
**Yu Guo, MSc**	Fuwai Hospital Chinese Academy of Medical Sciences, Beijing, China	Major role in the acquisition of data
**Yuanjie Pang, PhD**	Department of Epidemiology & Biostatistics, School of Public Health, Peking University, Beijing, China	Analysis or intepretation of data
**Dianjianyi Sun, PhD**	Department of Epidemiology & Biostatistics, School of Public Health, Peking University, Beijing, China	Analysis or intepretation of data
**Pei Pei, MSc**	Chinese Academy of Medical Sciences, Beijing, China	Major role in the acquisition of data

Appendix 1 *(continued)*

Name	Location	Contribution
**Ling Yang, PhD**	Medical Research Council Population Health Research Unit at the University of Oxford; Clinical Trial Service Unit & Epidemiological Studies Unit, Nuffield Department of Population Health, University of Oxford, UK	Major role in the acquisition of data
**Yiping Chen, DPhil**	Medical Research Council Population Health Research Unit at the University of Oxford; Clinical Trial Service Unit & Epidemiological Studies Unit, Nuffield Department of Population Health, University of Oxford, UK	Major role in the acquisition of data
**Huaidong Du, PhD**	Medical Research Council Population Health Research Unit at the University of Oxford; Clinical Trial Service Unit & Epidemiological Studies Unit, Nuffield Department of Population Health, University of Oxford, UK	Major role in the acquisition of data
**Hao Wang, MSc**	NCDs Prevention and Control Department, Zhejiang CDC, Hangzhou, China	Major role in the acquisition of data
**M. Sofia Massa, PhD**	Clinical Trial Service Unit & Epidemiological Studies Unit, Nuffield Department of Population Health, University of Oxford, UK	Drafting/revision of the manuscript for content, including medical writing for content
**Derrick Bennett, PhD**	Clinical Trial Service Unit & Epidemiological Studies Unit, Nuffield Department of Population Health, University of Oxford, UK	Drafting/revision of the manuscript for content, including medical writing for content
**Robert Clarke, FRCP**	Clinical Trial Service Unit & Epidemiological Studies Unit, Nuffield Department of Population Health, University of Oxford, UK	Drafting/revision of the manuscript for content, including medical writing for content
**Junshi Chen, MD**	China National Center for Food Safety Risk Assessment, Beijing, China	Study concept or design
**Zhengming Chen, DPhil**	Clinical Trial Service Unit & Epidemiological Studies Unit, Nuffield Department of Population Health, University of Oxford, UK	Study concept or design
**Jun Lv, PhD**	Department of Epidemiology & Biostatistics, School of Public Health, Peking University; Peking University Center for Public Health and Epidemic Preparedness & Response; Key Laboratory of Molecular Cardiovascular Sciences, Peking University, Ministry of Education, Beijing, China	Drafting/revision of the manuscript for content, including medical writing for content; Study concept or design
**Liming Li, MPH**	Department of Epidemiology & Biostatistics, School of Public Health, Peking University; Peking University Center for Public Health and Epidemic Preparedness & Response, Beijing, China	Major role in the acquisition of data; Study concept or design; Other

Appendix 2 Coinvestigators

Coinvestigators are listed at links.lww.com/WNL/B917
